# Dual targeting of glioblastoma multiforme with a proteasome inhibitor (Velcade) and a phosphatidylinositol 3-kinase inhibitor (ZSTK474)

**DOI:** 10.3892/ijo.2013.2205

**Published:** 2013-12-02

**Authors:** LEHANG LIN, DARIA GAUT, KAISHUN HU, HAIYAN YAN, DONG YIN, H. PHILLIP KOEFFLER

**Affiliations:** 1 Guangdong Provincial Key Laboratory of Malignant Tumor Epigenetics and Gene Regulation, Medical Research Center, Sun Yat-Sen Memorial Hospital, Sun Yat-Sen University, Guangzhou 510120, P.R. China;; 2 Division of Hematology/Oncology, Cedars-Sinai Medical Center, UCLA School of Medicine, Los Angeles, CA 90048, USA;; 3 Cancer Science Institute of Singapore, National University of Singapore, Singapore 117599, Republic of Singapore

**Keywords:** proteasome inhibitor, phosphatidylinositol 3-kinase inhibitor, glioblastoma multiforme, Velcade, ZSTK474

## Abstract

Proteasome inhibitors have been proven to be effective anticancer compounds in many tumor models, including glioblastoma multiforme (GBM). In this study, we found that the proteasome inhibitor Velcade (PS-341/bortezomib) caused GBM cell death while simultaneously activating the PI3K/Akt pathway. Therefore, we sought to investigate if the PI3K inhibitor ZSTK474 would enhance the effectiveness of Velcade in anticancer therapy. Two GBM cell lines were used to detect the effects of Velcade and ZSTK474 alone or in combination *in vitro*. The combination of Velcade and ZSTK474 synergistically inhibited the proliferation of GBM cell lines. Cell apoptosis was increased when exposed to Velcade and ZSTK474 in combination as shown by Annexin V analysis. Treatment with both drugs led to downregulation of the p-Akt, p-4EBP1 and p-mTOR proteins as determined by western blot analysis. The anticancer ability of Velcade for glioblastoma multiforme was, therefore, enhanced by combination with the PI3K pathway inhibitor ZSTK474 in glioblastoma multiforme.

## Introduction

The ubiquitin-proteasome pathway is responsible for degrading many of the short-lived regulatory proteins which govern cell division, growth, activation, signaling and transcription (1). Proteasome inhibition is a novel approach to the treatment of solid tumors (2). Velcade (PS-341/bortezomib) is a dipeptidyl boronic acid inhibitor with high specificity for the proteasome and the first proteasome inhibitor evaluated in clinical trials (1,3) and approved by the US Food and Drug Administration (FDA). We previously found that Velcade had profound effects on the growth and apoptosis of GBM cells (4). However, in this study, we found that Velcade simultaneously caused an increase in P-Akt and left mTOR signaling active in GBM cells.

Glioblastoma multiforme (GBM) is the most common primary brain tumor in adults and known as having one of the worst prognoses of all cancers. Successful treatment for GBM is rare. The median survival for patients is 10–12 months, despite aggressive surgical approaches, optimized radiation therapy regimens and cytotoxic chemotherapies (5).

The PI3K/Akt pathway in GBM cells is highly active, making it an ideal target for cancer therapy (5). Phosphatidylinositol 3-kinases (PI3Ks) are a class of lipid kinases active in signal transduction that generate phosphatidylinostiol-3,4,5-triphosphate (PIP_3_) by phosphorylating phosphatidylinositol-4,5-bisphosphate (6). They are involved in various cellular processes, including cell proliferation, adhesion, survival and motility, all of which are critical for tumorigenesis (7). Mutation and/or amplification of PI3K genes have been reported in many cancer cells, including glioblastoma (7,8).

PI3Ks are activated by receptor tyrosine kinases (RTKs). GBM cells commonly overexpress the oncogene epidermal growth factor receptor (EGFR) and the platelet derived growth factor receptor (PDGFR), both of which are the most common RTKs (9). Downstream of these receptors, the tumor suppressor gene PTEN, is also commonly mutated, further promoting the activation of PI3K/AKT pathway (5). Activation of PI3K pathway members, such as P-PI3k, P-p70^26k^ and P-Akt, has been proven to significantly reduce overall survival times when gliomas of all grades are considered (10). Many inhibitors of PI3K have been extensively studied, such as wortmannin and LY294002. ZSTK474 [2-(2-difluoromethylbenzimidazol-1-y1)-4,6-dimorpholino-1,3,5-triazine] is a novel PI3K inhibitor.

In the present study, the synergistic anti-glioma activity of Velcade and ZSTK474 was examined using two GBM cell lines. Treatment with both drugs inhibited proliferation and increased apoptosis of GBM cells. Detrimental proteins for therapy, such as P-Akt, P-4EBP1 and P-mTOR, were downregulated in the presence of both drugs. Taken together, treatment with the combination of Velcade and ZSTK474 was highly effective against GBM and might have a role in the future therapy for this aggressive disease.

## Materials and methods

### Glioma cell lines

Human GBM cell lines U87 and U118 were maintained in Dulbecco’s modified Eagle’s medium (Gibco, BRL) with 10% fetal calf serum (Gemini Bio-Products, Calabasas, CA, USA). Aliquots were cryopreserved in liquid nitrogen, and one aliquot of cells was kept in culture and grown to confluence. All cells were incubated at 37°C in 5% CO_2_.

### Chemicals

Proteasome inhibitor Velcade, obtained from Millennium Pharmaceuticals (Cambridge, MA, USA), was reconstituted with normal saline USP/EP at a stock concentration of 10^−4^ M and stored at −20°C. PI3K inhibitor ZSTK474, obtained from Selleckchem (Houston, TX, USA), was dissolved in DMSO at a stock concentration of 5×10^−3^ M and stored at −20°C. Fresh dilutions of media were made for each experiment.

### Cell growth inhibition

Cells were placed into 96-well plates at 5.0×10^3^ cells/well and respectively treated with single agent alone or their combination for 72 h. Cell proliferation was measured by MTT assay. Briefly, 20 *μ*l MTT solution (5 mg/ml) was added into each well for the last 4 h. Absorbance was measured at 570 nm using a spectrophotometer (Roche Molecular Biochemicals, Basel, Switzerland). Cell growth was determined in each group and compared with that of the untreated cells.

### Western blot analysis

Cells were harvested for total cell lysates with RIPA buffer (1% Nonidet P-40, 0.5% sodium deoxycholate, 0.1% SDS, 50 mM Tris-HCl, pH 7.5) containing a mixture of protease inhibitors (Roche Diagnostics GmbH, Mannheim, Germany) as well as 1 mM NaF and 1 mM NaVO_4_. Cell lysates were centrifuged at 12,000 rpm for 20 min at 4°C. Supernatant was collected, and the protein concentration was measured. The lysates (30 *μ*g) were denatured in the sample buffer (10% glycerol, 5% B-mercaptoethanol, 2.3% SDS, 62.5 mM Tris-HCl, pH 6.8) by boiling and then subjected to 4–15% SDS-PAGE followed by electrotransfer to polyvinylidene diflouride membrane. The immune complexes were visualized with Supersignal West Pico Chemiluminescent Substrate or Supersignal west dura extended duration substrate (Pierce, Rockford, IL, USA) and normalized by internal control (GAPDH). Antibodies against phosphorylated Akt (p-Akt), phosphorylated 4E-BP1 (p-4EBP1) and phosphorylated mTOR (p-mTOR) were bought from Cell Signaling Technology Inc. (MA, USA). Antibodies against cyclin D1, PARP and GAPDH were obtained from Santa Cruz Biotechnology Inc. (Santa Cruz, CA, USA).

### Assessment of apoptosis

Annexin V assays (Annexin V-FITC Apoptosis Detection kit; Pharmingen, San Diego, CA, USA) were performed according to the manufacturer’s instructions. Briefly, cells were harvested after exposure to drugs, washed twice with PBS, resuspended in 1X binding buffer at a concentration of 1×10^6^ cells/ml, incubated with FITC-conjugated Annexin V and PI for 15 min and measured by FACScan (Becton-Dickinson).

## Results

### Velcade activates the PI3K/Akt pathway in GBM cells

**Figure 1.**
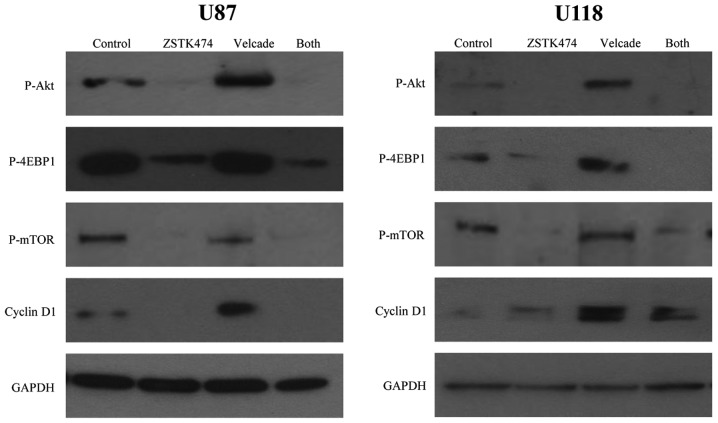
Effects of Velcade and ZSTK474 on expression of proteins central to the PI3K/Akt pathway in GBM cell lines. U87 and U118 were cultured for 24 h with Velcade (100 nM), ZSTK474 (2.5 *μ*M), or both simultaneously. Lysates were made and subjected to western blot analysis for P-Akt, P-mTOR, P-4EBP1 and cyclin D1 as well as GAPDH loading control.

The effectiveness of Velcade in causing growth arrest and apoptosis in GBM cells has been proven (4), but interestingly, we found that Velcade could stimulate the cell survival pathway PI3K/Akt simultaneously. Using western blot analysis, we found that Velcade could induce phosphorylation of Akt, 4E binding protein-1 (4EBP1) and mammalian target of rapamycin (mTOR) in both GBM cell lines compared to the control. As shown in Fig. 1, the amount of P-Akt was increased in cells treated with Velcade compared to the control. Furthermore, mTOR signaling was activated in the presence of Velcade as the amount of P-4EBP1 and P-mTOR was comparable to the control.

### Synergistic effects of Velcade and ZSTK474 on GBM cell line

To study whether the inhibition of the PI3K/Akt signaling pathway could enhance the Velcade-induced growth arrest of GBM cells, we treated cells with both Velcade and the PI3K inhibitor ZSTK474. When adding ZSTK474 (2.5 *μ*M, 24 h), the phosphorylation of Akt, 4EBP1 and mTOR was markedly decreased in both cell lines (Fig. 1). Cyclin D1, another protein central to cell proliferation in the PI3K/Akt pathway, was also upregulated in the presence of Velcade alone but downregulated when exposed in both drugs (Fig. 1).

**Figure 2.**
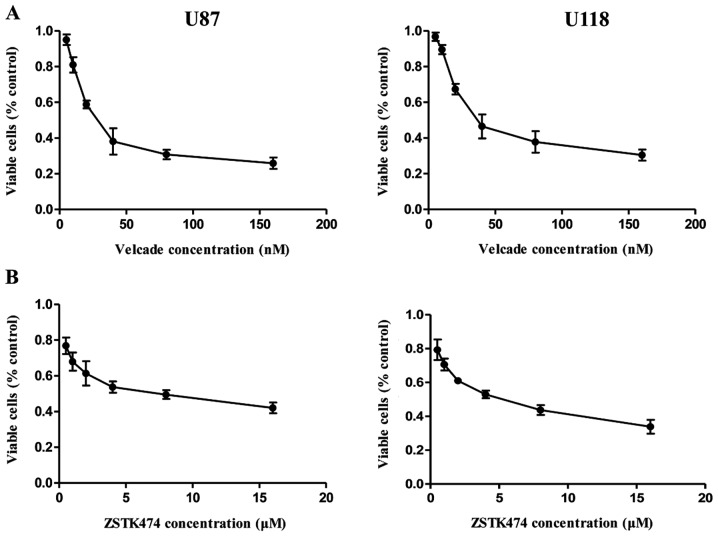
Effects of Velcade and ZSTK474 on GBM cell proliferation. Velcade dose-dependently decreased U87 and U118 cell growth after 72 h, with an IC_50_ of 40 and 54 nM (A). ZSTK474 dose-dependently inhibited growth with an IC_50_ of 7 and 5 *μ*M, respectively (B). The cell viability was measured by MTT assay and presented as a percent of the control. The IC_50_ values were calculated using the CalcuSyn software.

We also examined the effects of Velcade and ZSTK474 on the proliferation of GBM cells in liquid culture using the MTT (3-(4,5-dimethylthiazol-2-yl)-2,5-diphenyl tetrazolium bromide) assay. Two cell lines were treated with serial dilutions of Velcade or ZSTK474 alone for 72 h. Based on the growth inhibition assay, IC_50_ of Velcade and ZSTK474 was calculated to be 40 nM and 7 *μ*M in U87 cells, and 54 nM and 5 *μ*M in U118 cells (Fig. 2).

**Figure 3.**
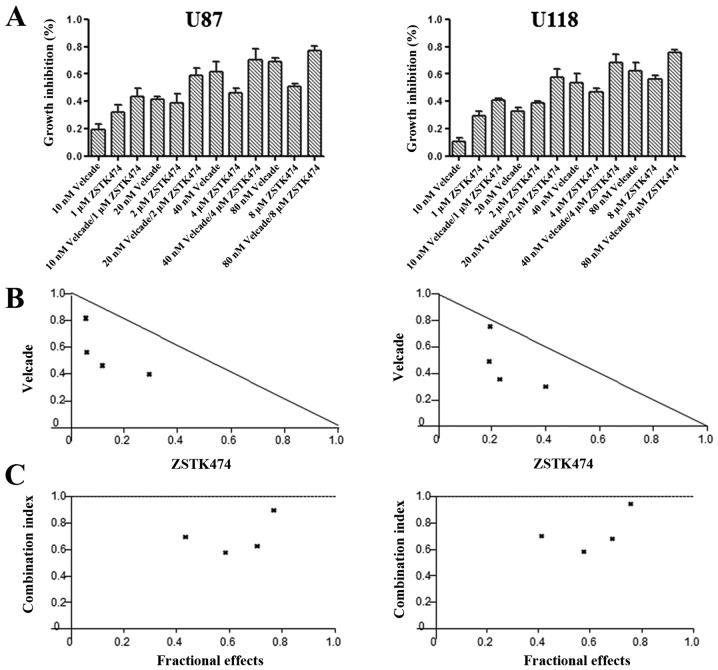
Synergistic effects of Velcade and ZSTK474 on GBM cell lines. Combination of Velcade and ZSTK474 at a fixed ratio synergistically inhibited cell growth (A). Isobologram analysis of all of the combination treatment groups fell below the synergistic threshold (B). Combination index (CI) values extrapolated from isobole analysis were <1 (C), altogether strongly indicating the synergistic effects of Velcade and ZSTK474 on GBM cells.

To evaluate the combined effects of Velcade and ZSTK474, cell lines were individually exposed to both drugs at a fixed ratio simultaneously for 72 h spanning the IC_50_ of each drug. The synergistic cytotoxicity was demonstrated by MTT assay (Fig. 3A), and the level of synergy at each dose combination was further validated by isobole analysis and CIs (Fig. 3B and C). The CI values of combined treatment with 10 nM Velcade and 1 *μ*M ZSTK474, 20 nM Velcade and 2 *μ*M ZSTK474, 40 nM Velcade and 4 *μ*M ZSTK474, 80 nM Velcade and 8 *μ*M ZSTK474 were 0.696, 0.576, 0.628 and 0.894 in U87 cells, and 0.702, 0.585, 0.682 and 0.949 in U118 cells, respectively. All of these values were well <1, suggesting high degree of synergism between both drugs.

### Velcade and ZSTK474 induces more apoptosis on GBM cells than either drug alone

**Figure 4.**
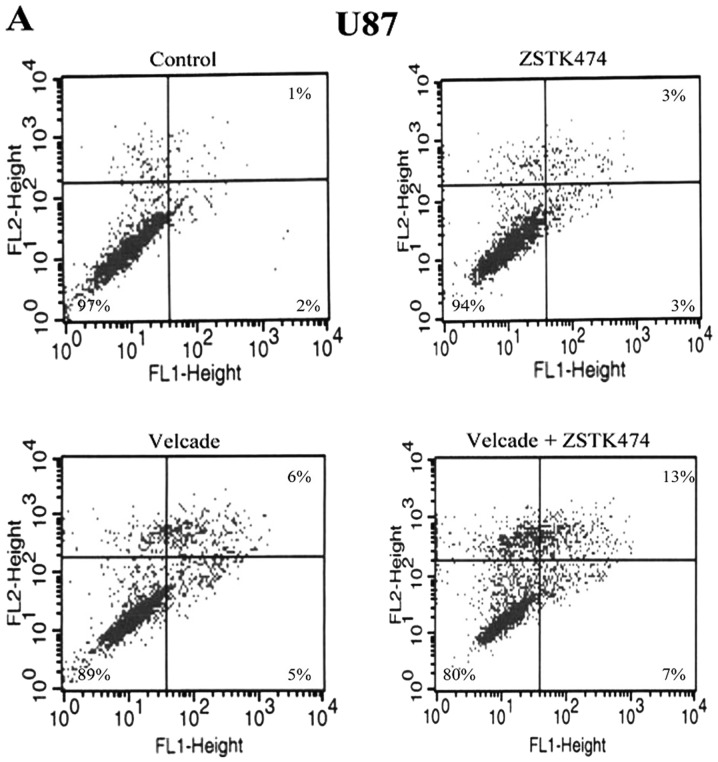

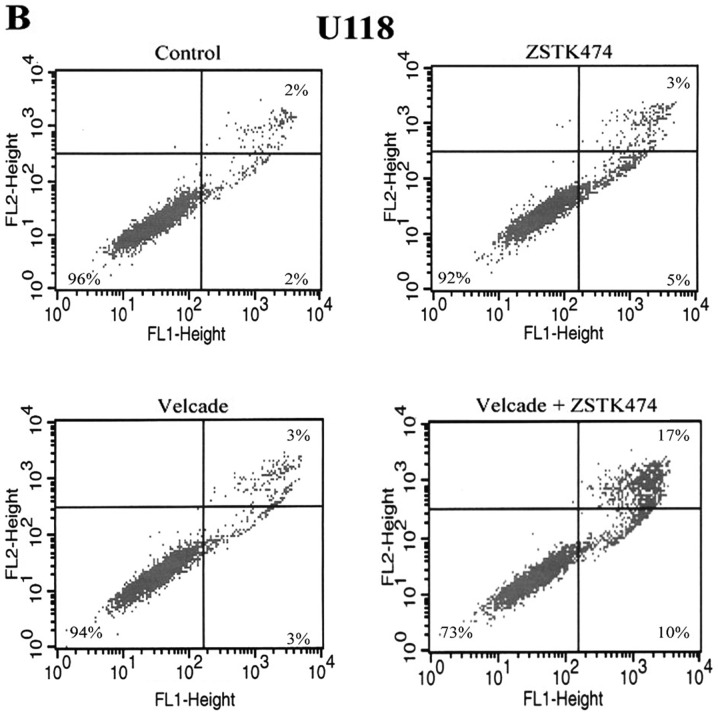
Apoptotic analysis of GBM cell lines treated with Velcade and ZSTK474. GBM cell lines were cultured for 24 h with 100 nM Velcade, 2.5 *μ*M ZSTK474, or both simultaneously. Apoptosis was assessed by Annexin V (FL-1) and PI (FL-3) staining and FACS analysis. Three experiments were carried out for each cell line and the results were similar. Representative experiments are shown.

In order to investigate whether the synergistic cytotoxicity was related to apoptosis, GBM cell lines were treated with 100 nM Velcade and 2.5 *μ*M ZSTK474 alone or their combination simultaneously for 24 h, and stained with Annexin V/PI. Apoptosis was determined by flow cytometry, including early apoptotic cells (Annexin V-positive, PI-negative) and late apoptotic cells (Annexin V- and PI-positive). The percentage of apoptotic cells in the treatment of Velcade and ZSTK474 alone or their combination was 11, 6 and 20% in U87 cells, and 6, 8 and 27% in U118 cells (Fig. 4), indicating that more apoptosis was caused in the presence of both drugs.

**Figure 5.**
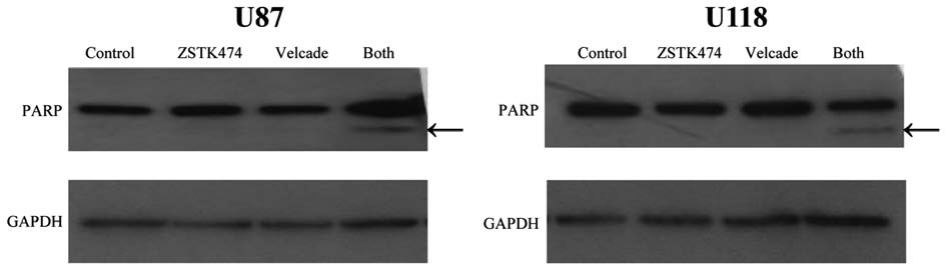
Effects of Velcade and ZSTK474 on the expression of apoptotic-related protein PARP in GBM cell lines. U87 and U118 were cultured for 24 h with Velcade (100 nM), ZSTK474 (2.5 *μ*M), or both simultaneously. Lysates were made and subjected to western blot analysis for poly(ADP-ribose) polymerase (PARP), generating a C-terminal 85-kDa apoptotic fragment when cells were treated with both drugs (arrow).

Immunoblotting analysis of the apoptotic-related protein poly(ADP-ribose) polymerase (PARP) showed that the C-terminal 85 kDa PARP apoptotic fragment was generated in each cell line when treated with both Velcade and ZSTK474 (Fig. 5).

## Discussion

Proteasome inhibitors are emerging as a promising class of agents in the treatment of cancers (1,2). In particular, the proteasome inhibitor Velcade (PS-341/bortezomib) has been in clinical trials for many years for cancer patients with advanced diseases, mostly multiple myeloma and mantle cell lymphoma (3,11). Based on positive preclinical and clinical studies, Velcade was approved by the US Food and Drug Administration (FDA) for clinical use for multiple myeloma treatment in 2008.

We have previously proven the effectiveness of Velcade in causing growth arrest and apoptosis in GBM cells (4). However, we recently discovered that in GBM Velcade also activated the PI3K/Akt pathway, which is known to increase tumorigenesis and is involved in cell proliferation (12). Through western blot analysis, we found that Velcade caused upregulation of P-Akt and left the mTOR signaling still active (Fig. 1).

Thus, we assumed that blocking the PI3K/Akt pathway might lead to the enhancement of the anticancer ability of Velcade. In order to deactivate the PI3K/Akt pathway, we used the novel PI3K inhibitor ZSTK474 in conjunction with Velcade. ZSTK474 has previously been proven to suppress tumor growth in GBM (13,14). With the presence of ZSTK474, P-Akt, P-4EBP1 and P-mTOR were down-regulated. Cyclin D1 was also upregulated in the presence of Velcade alone but downregulated with the combination of both drugs (Fig. 1).

In order to prove the synergistic effects of Velcade and ZSTK474, cell proliferation was examined by MTT assay. As the CI values of each dose combination were <1 (Fig. 3C), synergism was observed between the two drugs. Annexin V assays were also performed to study the induction of cell apoptosis, and an increase in apoptosis was observed in the presence of both drugs.

In conclusions, we demonstrated that Velcade and ZSTK474 was a synergistic drug combination that had profound effects on GBM cells. This was accompanied by increased apoptosis and decreased activation of many proteins central to the PI3K/Akt pathway. These findings suggested that combination treatment of Velcade and ZSTK474 might have a niche in the future therapy for glioblastomas.

## Abbreviations:

GBM: glioblastoma multiforme
PI3K: phosphoinositide 3-kinase
PARP: poly(ADP-ribose) polymerase
MTT: 3-(4,5-dimethylthiazol-2-yl)-2,5-diphenyl tetrazolium bromide

## References

[1] Adams J, Palombella VJ, Elliott PJ (2000). Proteasome inhibition: a new strategy in cancer treatment. Invest New Drugs.

[2] Yamamura M, Hirai T, Yamaguchi Y (2010). Proteasome inhibitor. Nihon Rinsho.

[3] Chen D, Frezza M, Schmitt S, Kanwar J, Dou QP (2011). Bortezomib as the first proteasome inhibitor anticancer drug: current status and future perspectives. Curr Cancer Drug Targets.

[4] Yin D, Zhou H, Kumagai T (2005). Proteasome inhibitor PS-341 causes cell growth arrest and apoptosis in human glioblastoma multiforme (GBM). Oncogene.

[5] Mischel PS, Cloughesy TF (2003). Targeted molecular therapy of GBM. Brain Pathol.

[6] Vivanco I, Sawyers CL (2002). The phosphatidylinositol 3-Kinase AKT pathway in human cancer. Nat Rev Cancer.

[7] Samuels Y, Wang Z, Bardelli A (2004). High frequency of mutations of the PIK3CA gene in human cancers. Science.

[8] Shayesteh L, Lu Y, Kuo WL (1999). PIK3CA is implicated as an oncogene in ovarian cancer. Nat Genet.

[9] Krakstad C, Chekenya M (2010). Survival signalling and apoptosis resistance in glioblastomas: opportunities for targeted therapeutics. Mol Cancer.

[10] Chakravarti A, Zhai G, Suzuki Y (2004). The prognostic significance of phosphatidylinositol 3-kinase pathway activation in human gliomas. J Clin Oncol.

[11] Ruggeri B, Miknyoczki S, Dorsey B, Hui AM (2009). The development and pharmacology of proteasome inhibitors for the management and treatment of cancer. Adv Pharmacol.

[12] Luo J, Manning BD, Cantley LC (2003). Targeting the PI3K-Akt pathway in human cancer: rationale and promise. Cancer Cell.

[13] Dan S, Yoshimi H, Okamura M, Mukai Y, Yamori T (2009). Inhibition of PI3K by ZSTK474 suppressed tumor growth not via apoptosis but G0/G1 arrest. Biochem Biophys Res Commun.

[14] Kong D, Yamori T (2009). Advances in development of phosphatidylinositol 3-kinase inhibitors. Curr Med Chem.

