# [Corrigendum] Dual targeting of glioblastoma multiforme with a proteasome inhibitor (Velcade) and a phosphatidylinositol 3-kinase inhibitor (ZSTK474)

**DOI:** 10.3892/ijo.2025.5777

**Published:** 2025-07-16

**Authors:** Lehang Lin, Daria Gaut, Kaishun Hu, Haiyan Yan, Dong Yin, H. Phillip Koeffler

Int J Oncol 44: 557-562, 2014; DOI: 10.3892/ijo.2013.2205

Following the publication of the above article, an interested reader drew to the attention of the Editorial Office that GAPDH bands featured for the U87 cell line (left-hand panels) in Fig. 5 on p. 561 were strikingly similar to the GAPDH bands for the U118 cell line (right-hand panels) shown in Fig. 1 on p. 559, even though the experiments shown in these figures were performed under different experimental conditions. Upon examining their data, the authors have realized that Fig. 5 was presented incorrectly; specifically, the cell lines ('U87' and 'U118') in Fig. 5 were mistakenly labeled in reverse, and the GAPDH bands from the right-hand panels of Fig. 1 were inadvertently re-used in the left-hand panels of Fig. 5.

The authors have now corrected the cell line labels and replaced the GAPDH bands in the left-hand panels of Fig. 5 with alternative data from a repeated experiment. The revised version of Fig. 5 is shown below. It is important to note that this error did not affect the overall conclusions reported in the study. The authors are grateful to the Editor of *International Journal of Oncology* for allowing them this opportunity to publish a Corrigendum, and all the authors agree with its publication. Furthermore, the authors deeply apologize to the readership for any inconvenience caused.

## Figures and Tables

**Figure 5:**
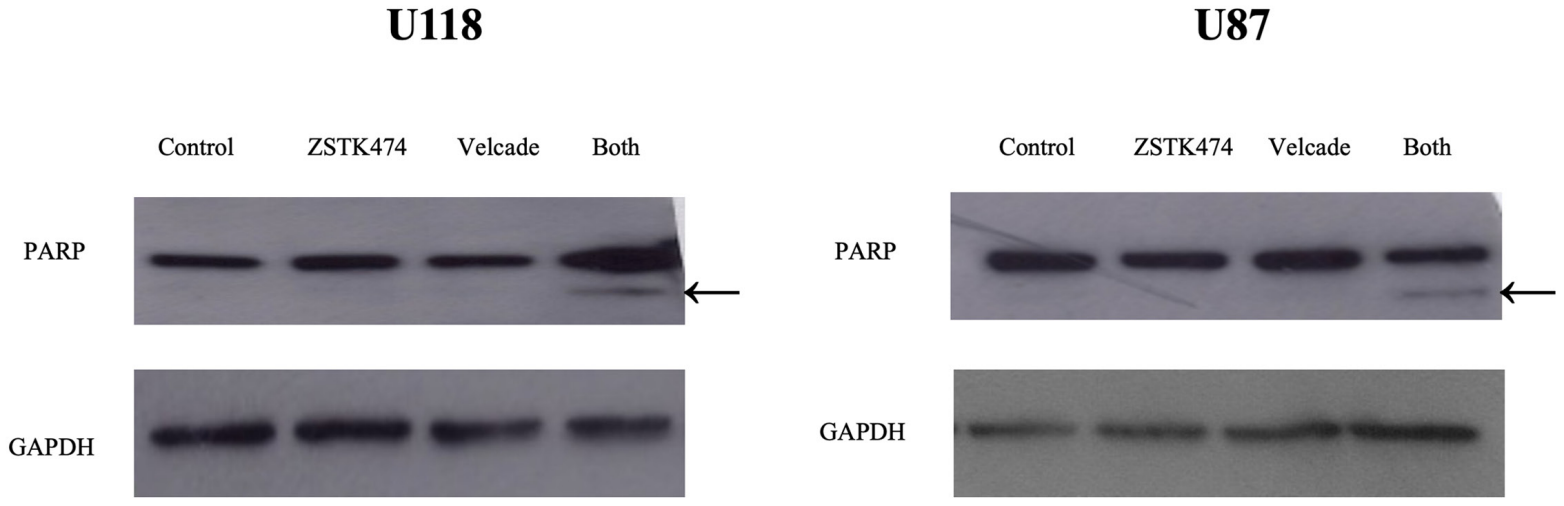
Effects of Velcade and ZSTK474 on the expression of apoptotic-related protein PARP in GBM cell lines. U118 and U87 cells were cultured for 24 h with Velcade (100 nM), ZSTK474 (2.5 μM), or both simultaneously. Lysates were made and subjected to western blot analysis for poly(ADP-ribose) polymerase (PARP), generating a C-terminal 85-kDa apoptotic fragment when cells were treated with both drugs (arrow).

